# Oral Peptide Vaccine against Hookworm Infection: Correlation of Antibody Titers with Protective Efficacy

**DOI:** 10.3390/vaccines9091034

**Published:** 2021-09-17

**Authors:** Ahmed O. Shalash, Luke Becker, Jieru Yang, Paul Giacomin, Mark Pearson, Waleed M. Hussein, Alex Loukas, Mariusz Skwarczynski, Istvan Toth

**Affiliations:** 1 School of Chemistry and Molecular Biosciences, The University of Queensland, St Lucia, QLD 4072, Australia; a.shalash@uqconnect.edu.au (A.O.S.); j.yang@uq.net.au (J.Y.); w.hussein@uq.edu.au (W.M.H.); i.toth@uq.edu.au (I.T.); 2 Centre for Molecular Therapeutics, Australian Institute of Tropical Health and Medicine, James Cook University, Cairns, QLD 4878, Australia; luke.becker@jcu.edu.au (L.B.); paul.giacomin@jcu.edu.au (P.G.); mark.pearson@jcu.edu.au (M.P.); alex.loukas@jcu.edu.au (A.L.); 3 School of Pharmacy, The University of Queensland, Woolloongabba, QLD 4102, Australia

**Keywords:** aspartic protease-1, hookworm, infection challenge, oral vaccine, peptide-based vaccine

## Abstract

Approximately 0.4 billion individuals worldwide are infected with hookworm. An effective vaccine is needed to not only improve the health of those affected and at high risk, but also to improve economic growth in disease-endemic areas. An ideal anti-hookworm therapeutic strategy for mass administration is a stable and orally administered vaccine. Oral vaccines are advantageous as they negate the need for trained medical staff for administration and do not require strict sterility conditions. Vaccination, therefore, can be carried out at a significantly reduced cost. One of the most promising current antigenic targets for hookworm vaccine development is the aspartic protease digestive enzyme (APR-1). Antibody-mediated neutralization of APR-1 deprives the worm of nourishment, leading to reduced worm burdens in vaccinated hosts. Previously, we demonstrated that, when incorporated into vaccine delivery systems, the APR-1-derived p3 epitope (TSLIAGPKAQVEAIQKYIGAEL) was able to greatly reduce worm burdens (≥90%) in BALB/c mice; however, multiple, large doses of the vaccine were required. Here, we investigated a variety of p3-antigen conjugates to optimize antigen delivery and establish immune response/protective efficacy relationships. We synthesized, purified, and characterized four p3 peptide-based vaccine candidates with: (a) lipidic (lipid core peptide (LCP)); (b) classical polymeric (polymethylacrylate (PMA)); and (c) novel polymeric (polyleucine in a branched or linear arrangement, BL_10_ or LL_10_, respectively) groups as self-adjuvanting moieties. BL_10_ and LL_10_ induced the highest serum anti-p3 and anti-APR-1 IgG titers. Upon challenge with rodent hookworms, the highest significant reduction in worm burden was observed in mice immunized with LL_10_. APR-1-specific serum IgG titers correlated with worm burden reduction. Thus, we provide the first vaccine-triggered immune response-protection relationship for hookworm infection.

## 1. Introduction

Hookworms are hematophagous parasites that infect half a billion individuals globally [1,2]. Hookworm infection is one of the most widespread but neglected tropical diseases. Its effects cause substantial economic losses to developing countries, in particular [3,4,5]. Despite the high efficacy of anthelmintic drugs, however, hookworms are developing resistance [6] and they do not prevent reinfection which is common in epidemic areas [7]. Therefore, an effective anthelmintic vaccine is urgently needed.

Early hookworm vaccines for veterinary purposes utilized whole parasite-based approaches. However, recombinant forms of larval antigens, e.g., Ancylostoma secretory protein-2 (ASP-2), caused strong allergenic responses in vaccinated human subjects [8,9]. X-ray irradiated/attenuated parasites-based vaccine offered protection against infection in dogs; however, it did not fully prevent reinfection, was difficult to produce, and had a short shelf life [10]. Thus, subsequent hookworm vaccine development efforts have instead focused on subunit vaccines and universal antigens existing in different hookworm species, such as *Necator americanus* (*Na*), *Ancylostoma duodenale* (*Ad*), and *Ancylostoma ceylanicum* (*Ay*) [3,4,5,11,12]. Among these antigens, cathepsin-D-like aspartic protease (APR-1), a crucial hookworm enzyme for digesting host hemoglobin, has been specifically targeted [13,14,15].

APR-1 is highly conserved in the three main hookworm species. Immunization with alum-adjuvanted, inactivated APR-1 resulted in 33% and 44% worm burden reductions when dog-*Ancylostoma caninum* and hamster-*Na* infection challenge models were used, respectively [16,17]. APR-1 was selected as one of two vaccine candidates (along with glutathione transferase-1) for the Sabin Vaccine Institute’s phase 1 clinical trials [18] as part of the HOOKVAC consortium initiative to reduce global hookworm burden [19].

However, in dogs at least, vaccination with recombinant APR-1 generated immune responses against several epitopes, including the highly immunogenic and non-neutralizing S_107_L epitope. Immunization with APR-1 could also potentially lead to the production of antibodies that recognize host proteins, as it is 38% and 46% identical to human pepsin and cathepsin-D, respectively. Furthermore, recombinant APR-1 is difficult to express and purify in sufficient yields for mass immunizations [15,20]. Monoclonal antibodies raised to recombinant *Na*-APR-1 that neutralized the enzyme’s ability to cleave substrates were shown to target the A_291_Y epitope (AGPKAQVEAIQKY) [15] and its extended version, p3 (TSLIAGPKAQVEAIQKYIGAEL) [20]. We have also demonstrated that the presence of β-sheet conformations in the epitope was crucial for the quality of generated epitope-specific antibodies, i.e., in their recognition of the native APR-1 protein [20]. Moreover, the p3 epitope is 100% conserved among APR-1 enzymes from major human hookworm species, as well as the canine hookworm (*Ancylostoma caninum*), the rodent hookworm (*Nippostrongylus brasiliensis*), and even other helminths, e.g., *Schistosoma mansoni*, *Schistosoma japonicum*, *Haemonchus contortus*, and *Caenorhabditis elegans* [21,22,23]. Whereas p3 is not conserved with human enzymes, e.g., cathepsin-D, renin, or pepsin [15], making p3 safer than the use of the protein antigen.

Peptide vaccines are often considered safer and more effective than whole protein-based alternatives. They are easily synthesized on massive scales to meet global demand, do not require cold-chain storage, are easily purified by liquid chromatography and are free of biological contaminants [24,25,26,27]. Peptide-based strategies are especially valuable for hookworm vaccines, as immune responses focused on the p3 peptide epitope can trigger the production of high levels of neutralizing antibodies, while avoiding the generation of antibodies against distracting, non-neutralizing epitopes, such as the S_107_L epitope in APR-1. Importantly, peptide-based vaccine immunogenicity can be increased dramatically with the addition of adjuvanting moieties [24,25], like chitosan [28] (e.g., polyacrylates [29,30]), lipids (e.g., lipid core peptide (LCP)) [31], or polyhydrophobic amino acids (e.g., polyleucine [32,33,34]). These systems have demonstrated superior immune-stimulating abilities comparable to, or exceeding, those of several commercial adjuvants [32] and provide highly improved oral vaccine immunogenicity [27,29,34,35].

Our initial studies on p3-based vaccines involved intraperitoneal immunization of BALB/c mice, where p3-LCP resulted in the production of anti-p3 IgG antibodies (p3-IgG) that recognized APR-1 and neutralized the active APR-1 enzyme in vitro [20,24]. Oral immunization of BALB/c mice using either p3-LCP or polyleucine-adjuvanted p3 along with the T-helper cell epitope P25 (KLIPNASLIENCTKAEL) [36] also resulted in the generation of anti-p3-IgG [34,35]. When orally immunized mice were challenged with *N. brasiliensis* third-stage larvae (L3) [37], high worm burden reductions were observed (90–100%). However, six doses (100 µg, each) were needed to induce sufficient levels of neutralizing antibodies, potentially due to in vivo degradation of the peptide antigens. In this study, we aimed to employ lower doses to distinguish subtle differences between new vaccine candidates and their protective efficacies in mice.

**Figure 1:**
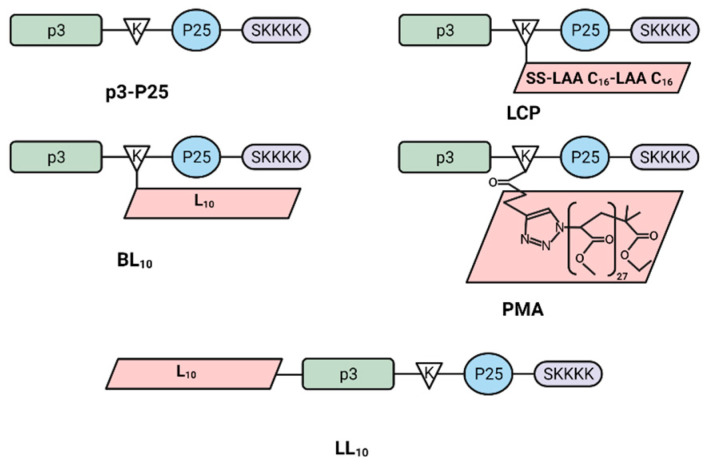
Peptide antigen (p3-P25) and vaccine candidate (LCP, BL_10_, LL_10_ and PMA) structures: p3 is an APR-1 B-cell epitope (TSLIAGPKAQVEAIQKYIGAEL), P25 is a T-helper epitope (KLIPNASLIENCTKAEL), and SKKKK is a solubilizing moiety.

We investigated whether novel vaccine candidates, designed to resist intestinal proteolysis, could trigger strong protective immune responses against hookworms with fewer doses. To facilitate comparison between the applied delivery systems, all vaccine candidates were produced in branched structure, although the polyleucine conjugate was also produced in linear structure (Figure 1). All candidates carried the APR-1 B-cell epitope (p3), a CD4-T cell epitope (p25), a solubilizing lysine-based unit (SKKKK), and a hydrophobic adjuvanting moiety. Following immunization of BALB/c mice with 4 × 100 µg doses of the vaccine candidates, antibody production, in vitro neutralization of APR-1 by anti-vaccine antibodies, and efficacy against challenge infection in vivo were investigated. Finally, we attempted to determine the correlation between immune responses and worm and egg burden reductions.

## 2. Materials and Methods

### 2.1. Materials

Fluorenylmethyloxycarbonyl (Fmoc-) protected L-amino acids and Rink amide MBHA resin were purchased from Novabiochem (Läufelfingen, Switzerland) and Mimotopes (Melbourne, VIC, Australia). 2-(1*H*-7-azabenzotriazol-1-yl)-1,1,3,3-tetramethyluronium hexafluorophosphate (HATU) was purchased from Mimotopes (Melbourne, VIC, Australia). *N,N* diisopropylethylamine (DIEA), *N,N*- dimethylformamide (DMF), trifluoroacetic acid (TFA), diethyl ether, dichloromethane (DCM), HPLC gradient-grade acetonitrile (MeCN), methanol and piperidine were purchased from Merck (Darmstadt, Germany). 4-Pentynoic acid was obtained from Novachem Pty Ltd. (Collingwood, VIC, Australia). Dialysis tubing (10,000 MWCO, snakeskin^®^, ID 22 mm) was purchased from Thermofischer (Scoresby, VIC, Australia). Triisopropylsilane (TIS), linear azide-terminated polymethyl acrylate polymer (2400 g/mol), and all other materials in analytical purity grade were obtained from Sigma-Aldrich (Castle Hill, NSW, Australia). 2-[(4,4-Dimethyl-2,6-dioxocyclohex-1-ylidene)ethylamino]-D,L-hexadecanoic Acid (Dde-C_16_ LAA) was synthesized as described previously [38].

Reverse-phase HPLC (Shimadzu Corp, Kyoto, Japan) with a Vydac C4 column was used for analysis and purification of the synthesized compounds. Attenuated total reflectance-Fourier transform infrared spectrophotometry (ATR-FTIR, Spectrum, PerkinElmer, Beaconsfield, UK) was used to evaluate the compounds’ secondary structures. Particle size distribution of the self-assembled nanoparticle compounds was evaluated using photon correlation spectroscopy (Zetasizer Nano, Malvern instruments, Worcestershire, UK). A fluorescence plate reader was used for evaluating in vitro APR-1 enzyme neutralization (BMG CLARIOStar^®^ fluorescent plate reader, BMG LABTECH, Cary, NC, USA). OriginPro 2020 software (OriginLab Corporation, Northampton, MA, USA) was used for mathematical data analyses.

### 2.2. Synthesis of Peptide Vaccines

All peptides were synthesized using Fmoc solid-phase peptide synthesis (SPPS) (Figure 1). Resin was swollen in DMF, then put through cycles of double-deprotection with 20% piperidine in DMF (1 × 5 min, then 1 × 10 min) and double-couplings to resin, using activated-protected amino acids (4.2 Eq), HATU (4.0 Eq) and DIEA (6.2 Eq) dissolved in DMF, for each amino acid (2 × 30 min). When the peptide sequences were complete, the N-termini were acetylated using acetic anhydride and DIEA in DMF (0.5:0.5:9.0). The resin was dried overnight in a desiccator, then the peptide was cleaved off using a 20 mL/g resin solution of TIS and MilliQ water in TFA (0.25:0.25:9.5). After cleavage, the peptides were precipitated using cold diethyl ether; extracted using a mixture of acetonitrile, MilliQ water, and TFA at a ratio of 90:10:0.1 for hydrophobic peptides, p3-K(L_10_)-P25-SK_4_ [BL_10_], L_10_-p3-K-P25-SK_4_ [LL_10_], p3-K(LCP)-P25-SK_4_ [LCP], or 45:55:0.1 for hydrophilic peptides, p3, p3-K(pentynoyl)-P25-SK_4_, and p3-K-P25-SK_4_ [p3-P25]; then freeze-dried to yield crude, dry peptide. The amino acid single code letter was used to describe the synthesized structures.

p3-P25. Yield: 62%. Molecular weight: 4909.9 g/mol. ESI-MS: [M + 4H]^4+^ *m*/*z* 1230.1 (calc.1228.5), [M + 5H]^5+^ *m*/*z* 982.9 (calc. 983.0), [M + 6H]^6+^ *m*/*z* 819.2 (calc. 819.3). HPLC *t*_R_: 20.60 min (0 to 100% solvent B, 40 min; C4 column), purity: 99%.

LCP. Yield: 49%. Molecular weight: 5632.9 g/mol. ESI-MS: [M + 5H]^5+^ *m*/*z* 1408.6 (calc. 1409.2), [M + 6H]^6+^ *m*/*z* 1127.6 (calc. 1127.6). HPLC *t*_R_: 27.29 min (0 to 100% solvent B, 40 min; C4 column), purity: 99%.

Pentynoylated-p3-P25. Yield: 41%. Molecular weight: 49890.0 g/mol. ESI-MS: [M + 4H]^4+^ *m*/*z* 1248.7 (calc. 1248.5), [M + 5H]^5+^ *m*/*z* 999.4 (calc. 999.0), [M + 6H]^6+^ *m*/*z* 832.5 (calc. 832.7), [M + 7H]^7+^ *m*/*z* 714.0 (calc. 713.9). HPLC *t*_R_: 21.40 min (0 to 100% solvent B, 40 min; C4 column), purity: 98%.

BL_10_. Yield: 50%. Molecular weight: 6083.5 g/mol. ESI-MS: [M + 4H]^4+^ *m*/*z* 1521.2 (calc. 1521.9), [M + 5H]^5+^ *m*/*z* 1217.9 (calc. 1217.7), [M + 6H]^6+^ *m*/*z* 1014.7 (calc. 1014.9), [M + 7H]^7+^ *m*/*z* 870.3 (calc. 870.1). HPLC *t*_R_: 27.27 min (0 to 100% solvent B, 40 min; C4 column), purity: 99%.

LL_10_. Yield: 50%. Molecular weight: 6043.2 g/mol. ESI-MS: [M + 4H]^4+^ *m*/*z* 1511.6 (calc. 1511.8), [M + 5H]^5+^ *m*/*z* 1209.5 (calc. 1209.6), [M + 6H]^6+^ *m*/*z* 1007.9 (calc. 1008.2), [M + 7H]^7+^ *m*/*z* 864.0 (calc. 864.3), [M + 8H]^8+^ *m*/*z* 756.4 (calc.756.4), [M + 9H]^9+^ *m*/*z* 672.9 (calc. 672.5). HPLC *t*_R_: 36.6 min (0 to 100% solvent B, 40 min; C4 column), purity: 98%.

Synthesis of PMA. A mixture of p3-K (pentynoyl)-P25-SK_4_ (6.0 mg, 1.2 mmole, 1.2 Eq) and azide-polymethyl acrylate polymer (2.4 mg, 1.0 mmole, 1.0 Eq) was dissolved in DMF (3 mL). Copper wire (50 mg) was added to the reaction mixture and the air was partially removed with nitrogen gas for 15–30 s. The reaction mixture was stirred at 50 °C for 14 h.

The PMA conjugate, p3-K(PMA)-P25-SK_4_ (Figure 1), in DMF solution was allowed to form nanoparticles by controlled antisolvent nanoprecipitation via syringe pump at a flow rate of 0.1 mL/hour into deionized water (water volume 4x greater than DMF volume), with stirring at 1000 RPM. The nanoparticles were purified from excess free/unconjugated peptide and DMF by dialysis (10,000 MWCO) in deionized water several times over three days; the compound was then freeze-dried for later use. Controlled antisolvent nanoprecipitation was conducted to produce fresh PMA conjugate nanoparticles prior to each immunization to ensure uniformity in particle size. A freeze-dried peptide-polymer conjugate (PMA) sample (5 mg) was analyzed using elemental microanalysis. The determined nitrogen to carbon ratio (N/C) provided the conjugation extent between nitrogen-rich peptide (N/C = 0.2850) and polymethylacrylate polymer (nitrogen-free, except for the azide group, N/C = 0.0312). The extent of conjugation was determined by plotting calculated theoretical N/C ratios, ranging from 0% (free polymer, N/C = 0.0312) to 100% conjugation (conjugate N/C = 0.205), followed by interpolation using the measured/found N/C ratio of the PMA conjugate (Figure S1). The synthesized PMA conjugate-measured N/C ratio (0.198) corresponded to a conjugation extent of 93%.

### 2.3. Peptide Vaccine Particle Size and Shape

Particle size distribution was evaluated by preparing a 0.2 mg/mL solution of each pure compound in PBS and measured using dynamic light scattering (DLS; Zetasizer Nano ZP) at 25 °C and an angle of 173°.

### 2.4. Transmission Electron Microscopy

Compounds were evaluated by transmission electron microscopy using a JEM-1010 microscope (JEOL LTD, Tokyo, Japan) at 100 kV. The images were analyzed using AnalySIS^®^ software (Megaview III, Munster, Germany).

### 2.5. In Vitro Stability against Proteolysis

Enzyme 1000 U solution was prepared from bovine trypsin (10,000 U/mg, Sigma-Aldrich, Castle Hill, NSW, Australia) with 1 mM CaCl_2_ in 1 mL PBS, as reported previously [39,40]. After preparation, 100 µL of enzyme solution was added to 400 µL of peptide solution (1.25 mg/mL) in PBS and incubated at 37 °C with shaking. To inhibit enzyme activity, 40 µL of TFA and acetonitrile mixture (1:2) was added to 60 µL aliquots of the mixture at each interval. The solution was analyzed by analytical HPLC with a calibration curve employing the compound’s peak at its characteristic retention time to determine intact compound concentration.

### 2.6. Secondary Structure of Peptide Vaccines

The compounds’ secondary structures were evaluated according to the methodology of Yang et al. [41]. Solutions of each compound in PBS (2 mg/mL) were prepared and placed on the diamond-attenuated total reflectance accessory of the ATR-FTIR, and absorbance spectra were collected from 500 and 4000 cm^−1^, in triplicate, at 25 °C and 4 cm^−1^ resolution. The first and second derivative of the absorbance spectra were generated and analyzed using Originpro 2020 software. The second derivative secondary structure component-peak assignment was also detected and quantified using Originpro 2020 software.

### 2.7. Oral Immunization of BALB/c Mice

Six groups of eight (6–8-week-old) female BALB/c mice (Animal Resources Centre, Perth, WA, Australia) were employed for the study. Naïve sera, saliva and feces were collected from each mouse two days prior to immunization. The mice were orally immunized via oral gavage with either PBS as a negative control, p3-P25/cholera toxin subunit B (CTB) as a positive control, or one of our vaccine candidates: LCP, PMA, BL10, or LL10. Vaccine doses contained 100 µg of antigen in 100 µL of PBS. Four immunizations were given to each group, with two-week intervals between doses, over six weeks, followed by an infection challenge by the end of the eighth week. An infection challenge was performed two weeks after the last immunization. Sample processing was as follows: sera were diluted 10-fold in PBS; saliva samples were undiluted; fecal samples (0.1 g) were homogenized in 1 mL PBS containing 0.05 *v/v*% tween 20 and 0.05 *w/v*% sodium azide.

### 2.8. Serological and Mucosal Immunogenicity Assays

Enzyme-linked immunosorbent assays (ELISA) were used to evaluate antibody titers of p3-P25, LCP, PMA, BL_10_, and LL_10_ using 96-well, flat bottom and high binding affinity plates (Sarstedt, Germany). The plates were coated with 100 µL of solution in carbonate buffer (pH 9.6) with either p3 epitope (1 µg/mL) or mutant APR-1_M74_ (0.5 µg/mL) expressed as previously described [15]. The plates were incubated at 37 °C for 90 min. PBS containing 0.05% tween 20 (PBST) was used as wash buffer. The plates were washed using deionized water, followed by PBST, then dried by tapping. PBST with 0.5% skim milk was added to the plates at a volume of 135 µL for wells in the first column, and 100 µL to all remaining wells. Then, 15 µL of 10-fold diluted serum from each mouse was added to first-column wells, and the plates were mixed. Serial dilutions (3-fold) were done along plate rows using a multichannel pipette (Biotools Pty Ltd., Loganholme, QLD, Australia) and an auto-ELISA system (Viaflo ASSIST, Integra Biosciences, Hudson, NH, USA). For fecal samples, 100 µL of PBST with 0.5% skim milk was added to each well. 50 µL of fecal extract was added to first-columns wells. The plates were mixed and then serially diluted (3-fold) along the rows. The plates were incubated at 37 °C for 90 min, then washed using deionized water and wash buffer, and dried by tapping. Secondary antibody solution (100 µL, 1/4000 diluted in PBST with 0.5% skim milk) of either goat anti-mouse IgG-HRP (Bio-Rad, Gladesville, NSW, Australia), goat anti-mouse IgA-HRP (Life Technologies, Mulgrave, VIC, Australia), goat anti-mouse IgG1-HRP (Bio-Rad, Gladesville, NSW, Australia), or goat anti-mouse IgG2a-HRP (Bio-Rad, Gladesville, NSW, Australia) was added to the wells depending on antibody isotype or subclass of interest. The plates were incubated again at 37 °C for 90 min, then washed using deionized water and wash buffer, and dried by tapping. Finally, peroxidase o-phenylenediamine dihydrochloride (OPD) substrate was applied for 15 min. The reaction was stopped using 100 µL of 1 N sulfuric acid and the absorbance of each well was measured using a SpectraMax 250 microplate reader (Molecular Devices, San Jose, CA, USA) at 450 nm. The titer was described as the lowest dilution that produced an absorbance above the mean absorbance of control wells (containing naïve mouse serum that was sampled pre-immunization) plus 3 standard deviations.

### 2.9. Enzyme Neutralization

The enzyme neutralization assay was designed based on the strategy reported by Pearson et al. [13,15,24] but modified to suit immune mice sera instead of purified neutralizing antibodies. A functional APR-1 wild-type (10 ng/50 µL) solution, expressed as described previously [13,15,42], in 50 mM sodium acetate pH 3.5 buffer was incubated with MoCAc-GKPILFFRLK, hemoglobin-substitute pre-fluorescence substrate (1 µM final concentration), in the presence or absence of immune mouse serum in the wells of Corning^®^ costar white opaque fluorescence 96-well plates. The reaction volume was adjusted to 100 µL, using acetate buffer pH 3.5. The sera from four mice per group were pooled to result in two pooled serum samples per group. A total of 5 µL of each stock was added to the enzyme and allowed to bind/neutralize for 30 min. The fluorescent substrate was added immediately prior to commencement of fluorescence measurement using SpectraMax 250 microplate reader (Molecular Devices, San Jose, CA, USA). The plate wells were temperature-controlled (37 °C) and measured for fluorescence intensity in 5-min cycles for 2 h, at an excitation wavelength of 330 nm. Signals were recorded at an emission wavelength of 390 nm using appropriate optic filters. The photomultiplier tube gain was set at 75%, with auto-adjusted focal height for optimum signal output. The kinetics of substrate proteolysis was monitored over the measurement period to compare relative inhibition efficacies of the different groups’ sera. Pooled immune sera relative fluorescence units (RFU) were presented as time-integral percentage increases in RFU values from the onset of the experiment.

### 2.10. Challenge Infection

All life stages of *N. brasiliensis* were maintained in Sprague Dawley rats. From these, an L3 larval suspension was prepared and each mouse was infected subcutaneously in the scruff with 750 larvae, as recommended for rat-adapted larvae infectivity in mice [37]. The mice were euthanized 7 days post-infection, and their intestines were isolated to conduct intestinal adult worm counts. Colon fecal contents were collected to conduct egg counts as described previously [34,35,37]. Briefly, fecal contents were weighed (with a range of 100–200 mg), and 4–10 mL of saturated salt solution was added. The feces were homogenized and the egg suspension was loaded into 300 µL-chambers of a counting slide, placed under a microscope, and counted twice. The number of eggs per gram of feces was determined using Equation (1), where V is the total volume of salt solution added in mL, and W is the feces weight in grams. Intestinal worm burden was determined by isolating the intestines from cecum to pylorus. The intestine was cut longitudinally and placed in a petri dish containing 5 mL sterile PBS, then incubated at 37 °C for 1 h. Adult hookworms were counted using a dissection microscope at 2×-magnification.

(1) eggg=(Count 1+Count 2) · 1000 · V600 · W

### 2.11. Statistical Analysis

Parametric one-way ANOVA with Tukey’s multiple comparison test was used to evaluate all assays. Non-parametric Mann Whitney U-tests were additionally used for challenge infection worm and egg burden analyses [34,35]. Significance was set at *p* < 0.05 (*), *p* < 0.01 (**), *p* < 0.001 (***), and *p* < 0.0001 (****).

### 2.12. Ethics Statement 

All animal experiments were approved by the James Cook University (JCU) Animal Ethics Committee (ethics number A2571). The animals were housed at JCU, and allowed free access to pelleted food and water, in accordance with the Queensland Animal Care and Protection Act.

## 3. Results

### 3.1. Synthesis and Nanoparticle Characterization

The final vaccine candidates and the vaccine components (p3-P25, LCP, pentynoylated-p3-P25, BL_10_, and LL_10_) were synthesized using peptide SPPS, purified by RP-HPLC and analyzed by RP-HPLC and ESI-MS (Supplementary Figure S1). Pentynoylated p3-P25 was further conjugated to azide-terminated linear polymethyl acrylate polymer to prepare the PMA compound. 

**Figure 2:**
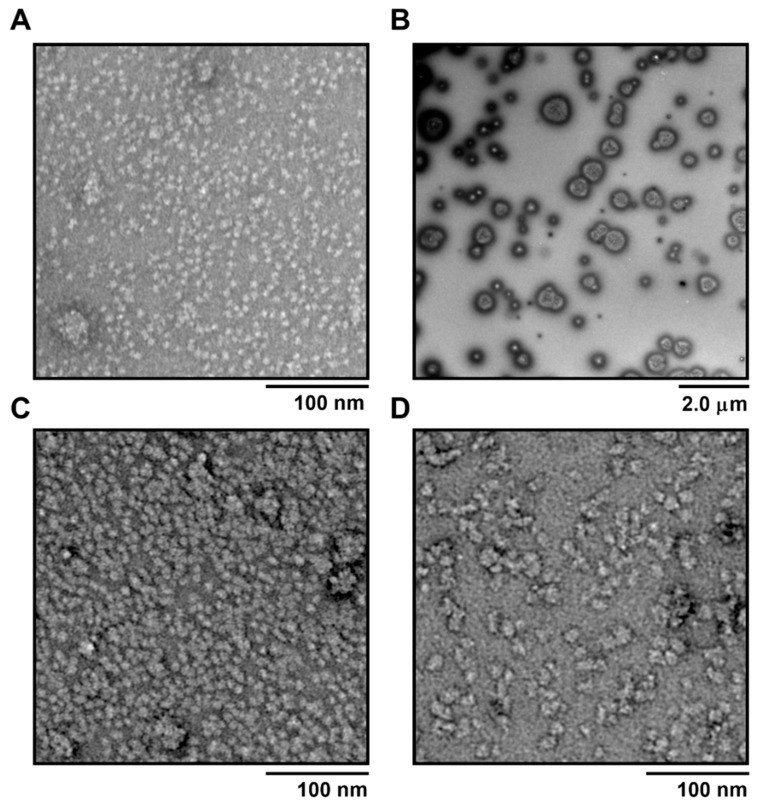
Transmission electron microscopy of self-assembled nanovaccine compounds: LCP (**A**), PMA (**B**), BL_10_ (**C**), and LL_10_ (**D**).

**Figure 3:**
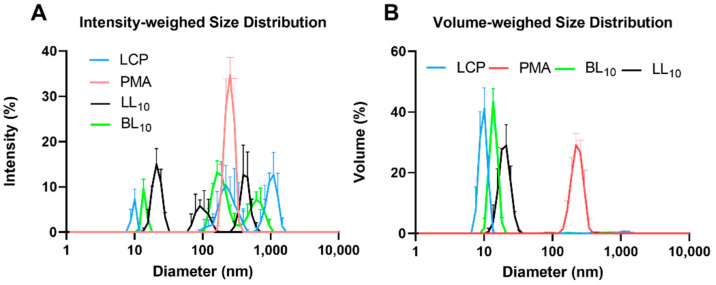
Conjugate particle sizes, as measured by dynamic light scattering, presented as: intensity (**A**) and volume distribution (**B**).

As the peptide conjugates were designed to possess both hydrophobic and hydrophilic moieties, they all self-assembled into nanoparticles. LCP, BL_10_ and LL_10_ formed mixtures of small nanoparticles (10 nm, 14 nm and 22 nm, respectively) and aggregates when analyzed by electron microscopy and DLS (Figure 2 and Figure 3). However, only small nanoparticles were observed by volume-weighted distribution (Figure 2 and Figure 3B). As expected, the presence of larger aggregates resulted in high polydispersity index (PDI) values for LCP (0.86 ± 0.1), BL_10_ (0.79 ± 0.2), and LL_10_ (0.36 ± 0.1). In contrast, PMA formed relatively large, but monodisperse, nanoparticles (230 nm, PDI = 0.211 ± 0.01). Nanoparticle sizes were also confirmed by TEM (Figure 2).

### 3.2. Secondary Structure

**Figure 4:**
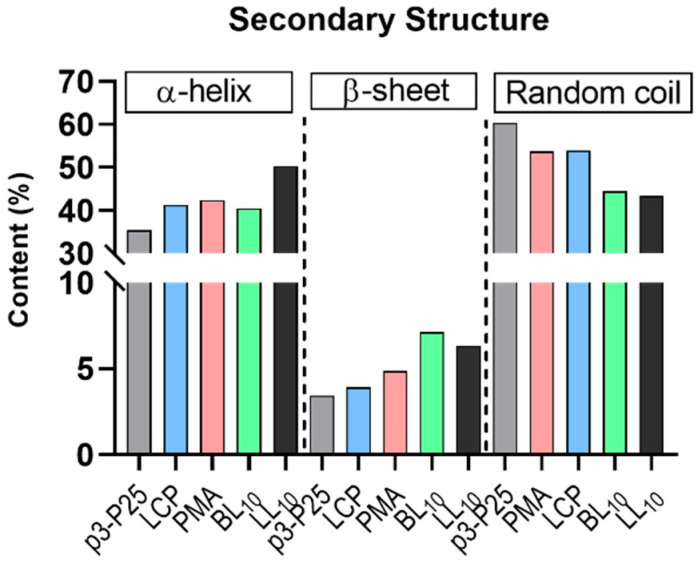
Secondary structure of p3-P25, LCP, PMA, BL_10_ and LL_10_, as determined by ATR-FTIR.

PMA nanoparticle size was similar to the light wavelength used in circular dichroism (CD) analysis and, therefore, caused CD spectrum flattening due to scattered radiation [43,44,45]. Therefore, we used ATR-FTIR to evaluate the secondary structure of all conjugates (Figure 4 and Figure S2). The control peptide, p3-P25, adopted a predominantly random coil structure (60%) and α-helical (35%) conformation, with very minor β-sheet contribution (3%). Polyleucine-adjuvanted compounds also had the tendency to form random coil and helical structures; however, β-sheet contribution significantly increased to 7% and 6% for BL_10_ and LL_10_, respectively. However, it is worth mentioning that LCP, PMA, BL_10_ and LL_10_ carry P25 (T-cell epitope) and adjuvanting moieties that affect the conformational content of each conjugate. This predicts potentially higher β-sheet contribution in p3 epitope conformation within the conjugates than for the whole conjugates. Furthermore, the quantitative secondary structure percentage values of non-polymeric peptide vaccines measured by circular dichroism spectropolarography, described previously [32], agree with ATR-FTIR results.

### 3.3. Enzymatic Stability In Vitro

**Figure 5:**
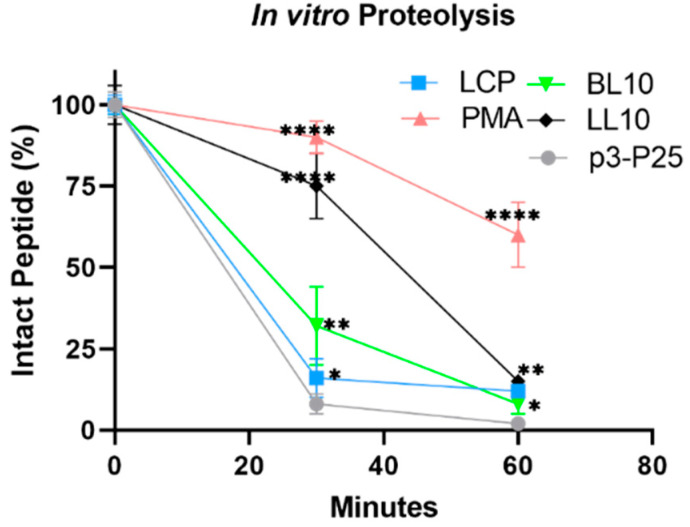
Stability of synthesized compounds against bovine trypsin in vitro proteolysis. Statistical analysis was performed using one-way ANOVA with Tukey’s multiple comparison test (*p* > 0.05; (*) *p* < 0.05; (**) *p* < 0.01; and (****) *p* < 0.0001).

The proteolytic stability of the peptide vaccines was investigated against bovine trypsin digestion at 37 °C (Figure 5). PMA stability was significantly higher (*p* < 0.0001) than that of all other vaccine compounds, with only 10% degradation observed after 30 min of incubation with trypsin. LL10 was also relatively resistant to proteolysis (25% degradation at 30 min, *p* < 0.0001); however, only 15% of the conjugate remained intact after 60 min. BL10 and LCP were more rapidly degraded by trypsin; however, their degradation was still significantly slower than p3-P25. 

### 3.4. Immunogenicity of the Conjugates

**Figure 6:**
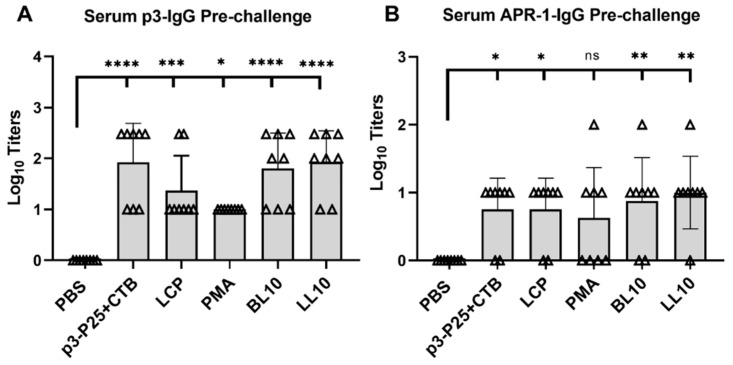
Serum p3-IgG (**A**) and APR-1-IgG (**B**) log10 titers from immunized and control mice. Each point represents an individual mouse; bars represent the average antigen-specific serum IgG antibody titers. Statistical analysis was performed using one-way ANOVA with Tukey’s multiple comparison test (ns, non-significant, *p* > 0.05; (*) *p* < 0.05; (**) *p* < 0.01; (***) *p* < 0.001; and (****) *p* < 0.0001).

**Figure 7:**
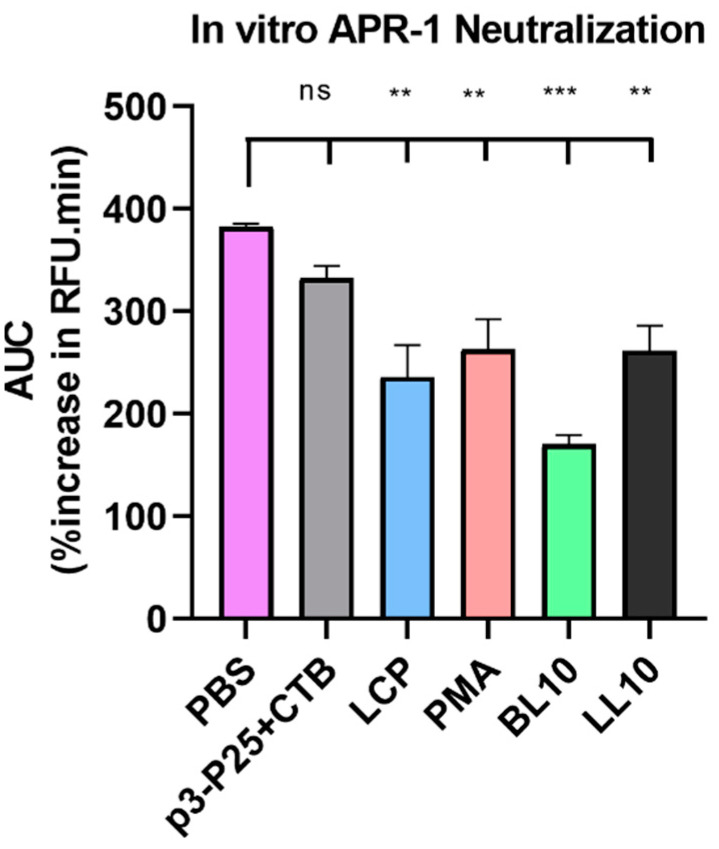
In vitro serum APR-1 enzyme neutralization test: integration of substrate-generated fluorescence (RFU) over 30 min per serum group. Fluorescent substrate digestion by APR-1 was inhibited by immune sera and compared to sera from the negative control group. Statistical analysis was performed using one-way ANOVA with Tukey’s multiple comparison test (ns, non-significant, *p* > 0.05; (**) *p* < 0.01; and (***) *p* < 0.001).

Antibody titers were evaluated using ELISA for mouse fecal extracts, saliva samples and serum (Figure 6). Mice immunized with LCP, PMA, BL10, and LL10 produced significantly higher p3-IgG titers compared to those in the PBS group (Figure 6A). However, only some of these antibodies recognized the parent protein (APR-1). In addition, IgA and IgG levels in the saliva and fecal extracts were low, IgG2a levels in the serum were undetectable, and only mice immunized with LL10 and BL10 generated detectable mucosal antibody responses (Figures S3 and S4). No significant level of APR-1-specific antibody production was detected in fecal samples, most likely due to the sensitivity limitations of the method (detectability of such low titer levels). Sera from all immunized mice, except for those in the p3/CTB group, were able to significantly reduce APR-1 enzymatic activity compared to sera from PBS-immunized mice (Figure 7). The mice were monitored on daily basis between immunizations, and they did not exhibit any adverse events or clinical signs of illness or toxicity.

### 3.5. Infection Challenge

**Figure 8:**
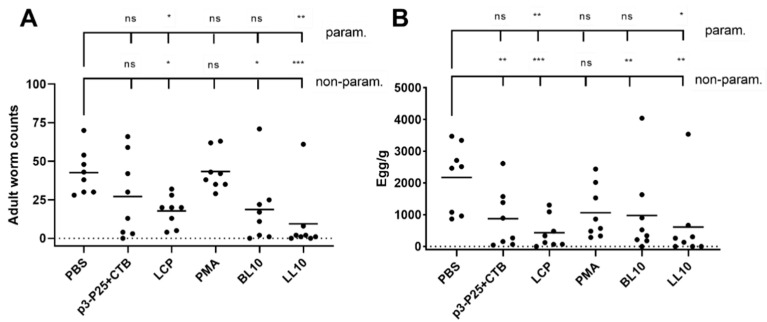
Infection challenge of BALB/c mice (*n* = 8/group) with 750 Nippostrongylus brasiliensis L3 larvae per mouse. Adult worm counts (**A**) and egg burden per gram of feces (**B**). Parametric (one-way ANOVA) and non-parametric (Mann Whitney U-test) tests were used to compare each immunized group against the negative control group. The significance was set at *p* < 0.05 (*), *p* < 0.01 (**), and *p* < 0.001 (***).

Mice were euthanized 7 days post-infection, and hookworms in the intestines and eggs in the feces were counted (Figure 8). Vaccination with LL10 and LCP resulted in significantly reduced parasite and egg numbers after challenge infection; BL10 also produced significant protective immune responses according to non-parametric analysis. 

## 4. Discussion

The immunogenicity of oral vaccines is affected by the interplay of three variables: oral delivery and uptake of nanoparticles, stability against enzymatic digestion, and intrinsic vaccine construct immunogenicity. Of the conjugates tested, only PMA demonstrated poor efficacy in the challenge study. Its conformational properties did not differ significantly from LCP, LL_10_ or BL_10_; however, PMA was very stable against enzymatic degradation and formed much larger particles than the other conjugates (Figure 2). It is worthy to mention that the trypsin cleavage sites were present only in p3-P25 and this peptide was degraded almost completely in less than 30 min upon treatment with trypsin. All other conjugates contained this peptide and consequently possessed identical bovine trypsin cleavage sites. Thus, the enzymatic cleavage was inhibited by the presence of adjuvanting moieties and not by the reduced number of cleavage sites. Nanoparticles in the range of 20–50 nm are usually considered to be the most immunogenic [25]; thus, PMA’s reduced efficacy might be correlated with its larger than optimal nanoparticle size. In other words, the larger particle size of PMA may have compromised its uptake by M-cells, delivery to underlying lymph nodes, and/or its intrinsic immunogenicity.

**Figure 9:**
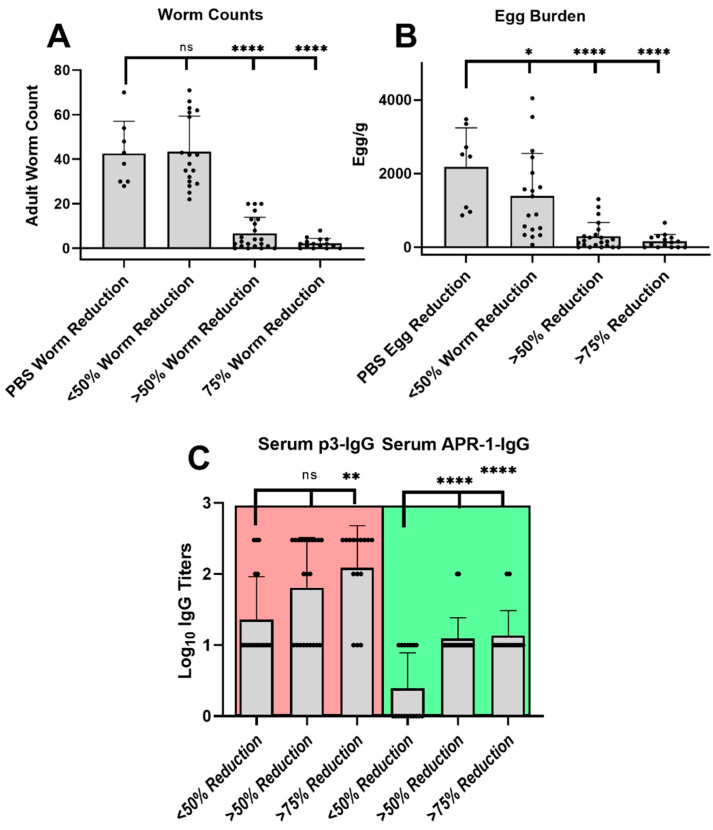
Correlation of antibody titers with protective efficacy for individual mice. The mice of all immunized groups were reshuffled according to their worm burden reduction; ‘High’ (>75%), ‘medium’ (>50%), and ‘low’ (<50%) worm burden reductions (**A**), to isolate the effective immune responses that distinguish the mice with superior protection. Egg burden reductions (**B**) corresponded with worm count categorization in panel (**A**). The correlation between worm burden reduction and serum anti-p3 IgG titers (p3-IgG) or serum anti-APR-1 IgG titers (APR-1-IgG) (**C**) among re-categorized/re-shuffled mice groups. Statistical analysis was performed using one-way ANOVA with Tukey’s multiple comparison test (ns, non-significant, *p* > 0.05; (*) *p* < 0.05; (**) *p* < 0.01; (***) *p* < 0.001; and (****) *p* < 0.0001).

Since in vivo-derived data were available for each mouse, we sought to categorize mice according to worm burden reduction. Three categories were formed: ‘high’ (>75% worm burden reduction), ‘moderate’ (50–75% worm burden reduction), and ‘low’ (<50% worm burden reduction). The percentage of reduction was calculated in comparison to worm levels in control mice immunized with PBS (Figure 9A). The same approach was applied to egg burden reduction (Figure 9B). Low worm and egg burden reductions were not substantially different from the PBS group, while moderate and high reduction burdens were significantly different (*p* < 0.0001).

We additionally analyzed the IgG titers generated by individual mice in correlation to worm burden reduction. High and medium worm burden reductions clearly correlated with APR-1-IgG titer levels when compared to mice with low reductions (*p* < 0.0001), while less significant differences (*p* < 0.01) were found when serum p3-IgG titers were used for the correlation (Figure 9C). This clearly suggests that anti-APR-1 serum antibodies underpin the protection mechanism resulting in worm reduction. The difference in significance levels between p3-IgG and APR-1-IgG demonstrates that the quality of antibody recognition of native epitope conformation is a critical factor in the efficacy of the immune response generated. 

Our categorization approach differentiated serum immune responses between vaccinated groups and pinpointed the most promising immune correlates of protection. In contrast, the correlation between mucosal antibody responses and worm burden reduction was, in general, not statistically significant (Supplementary Figure S5).

Immunizing mice groups with peptide vaccines resulted in different oral immunogenicity due to a variety of factors including their different self-assembled particle size (Figure 2 and Figure 3), stability against intestinal digestion (Figure 5), and intrinsic immunogenicity. The APR-1 neutralization capacity (Figure 7) of immune sera in vitro was shown to reduce worm burdens and egg counts in vivo in a similar manner (Figure 8 and Figure 9). Furthermore, the efficacy of humoral responses agrees with earlier findings showing that humoral responses are the primary means of protection against adult hookworms, where protection was passively conveyed via the transfer of immune sera to naïve rats or dogs [46,47]. This holds especially true here as a neutralizing B-cell epitope (p3) was employed as the constant core antigen in all of our vaccine constructs. Moreover, serum anti-p3 IgG titers measured between immunizations did not decrease, suggesting a lack of vaccine-induced immune tolerance (Figure S6). Further, mice sera did not generate anti-human cathepsin-D IgG titers or serum anti-APR-1 IgE titers (Figure S7), thus peptide vaccines resulted in protection without compromising safety via allergic or autoimmune responses. The lack of anti-cathepsin-D antibodies was expected since the peptide epitope (p3) is not conserved in human or murine cathepsin-D (Figure S8).

In summary, oral administration (4 × 100 µg) of self-assembled peptide nanovaccines of 10–25 nm in diameter induced antibody-based protective immune responses. These responses resulted in reduced intestinal worm burdens of 55–80% and fecal egg burden reductions of 55–85% in the BALB/c-*Nb* challenge infection model of human hookworm infection. Vaccine efficacy was reflected by a correlation between serum APR-1-specific IgG responses and worm and egg burden reductions. Linear polyleucine-adjuvanted compound (LL_10_) showed improved resistance against trypsin digestion in vitro, self-assembled into small-sized nanoparticles (22 nm) and generated a protective anti-APR-1 IgG response that resulted in the highest reduction of worm burden (80%) in the BALB/c-*Nb* challenge model.

## 5. Conclusions

In this study, we determined that APR-1-specific serum IgG is a significant immune correlate of protection against hookworm infection in vivo*,* in the absence of significant titers of mucosal/fecal APR-1-specific IgA/IgG. However, the generated IgG titers originated from gut-associated lymphoid tissue; therefore, local responses may have been stronger than peripheral (blood) responses, as these tissues are more concentrated around the infected intestinal sites. The IgG generated were dominated by the neutralizing IgG1 subclass, rather than IgG2, and protective efficacy did not significantly depend on antigen-specific salivary IgA/IgG. Moreover, we showed that fewer vaccine doses can still be effective; 4 × 100 µg oral immunizations with more stable, self-adjuvanted peptide, including LCP, LL_10_ and BL_10_, were adequate to significantly reduce worm burden by 56–78% (*p* < 0.05) in mice after *N. brasiliensis* larval challenge. In this experiment, the positive control (p3-P25 + CTB) was not able to significantly reduce worm burden (reductions of only 35%; *p* > 0.05) at the vaccine dose employed. Therefore, despite lower in vitro stability against trypsin digestion, BL_10_ resulted in similar serum titers to LL_10_. This suggests that BL_10_ is more immunogenic and readily taken up by gut-associated lymphoid tissue, resulting in similar serum anti-p3-IgG titers despite its lower stability against digestion. LCP, BL_10_ and LL_10_ compounds were more stable against enzymatic degradation, and were significantly more protective, compared to the positive control (p3-P25 + CTB). Our data further support the development of oral vaccines targeting enzyme neutralizing epitopes of *Na*-APR-1 to control human hookworm infection in disease-endemic countries. The findings also have potential implications for the development of vaccines against other gastrointestinal helminth infections of humans and domestic livestock animals.

## Data Availability

The data presented in this study are available in article and Supplementary Materials.

## Supplementary Materials

The following are available online at https://www.mdpi.com/article/10.3390/vaccines9091034/s1, Figure S1. Physicochemical characterization of vaccine candidates: MS-ESI spectra (A); elemental analysis (B); and HPLC chromatograms (C). Typical signal broadening can be observed for amphiphile conjugates, LL10, BL10 and LCP. Figure S2. An example of secondary structure determination by FTIR-ATR. Figure S3. Subclasses of serum p3-IgG titers: (A) serum p3-IgG1 titers, and (B) serum p3-IgG2a titers. These demonstrate that the main IgG subclass is of the neutralizing IgG1 type. The horizontal dashed line represents the starting dilution. Figure S4. Pre-challenge salivary p3-IgG (A) and p3-IgA (B), fecal p3-IgG (C), fecal p3-IgA (D), fecal APR-1-IgG (E), and fecal APR-1-IgA (F) log10 titers. Figure S5. Protective capacity of fecal p3-IgG/IgA (A), and salivary p3-IgG/IgA titers (B). Figure S6. Serum anti-p3 IgG titers after 1st immunization (A), 2nd immunization (B), 3rd immunization (C), and 4th immuniza-tion (D). Figure S7. Serum anti-human cathepsin-D IgG titers (A), and serum anti-APR-1 IgE titers were both undetectable using post-challenge mice sera. Figure S8 Protein sequences alignment of mouse cathepsin-D, Human cathepsin-, and Na-APR-1, orange highlighted sequence showing lack of similarity of p3 epitope (APR-1) to corresponding sequences in mouse or human cathepsin-D sequences. Mouse cathepsin-D (UniProt P18242) sequence is 89% identical to human cathepsin (UniProt P07339), and only 46% identical to hookworm Na-APR-1 enzyme sequence (UniProt Q9N9H3) using CLUSTALW.
