# Supplementary material for: Oral Peptide Vaccine against Hookworm Infection: Correlation of Antibody Titers with Protective Efficacy
Source: Vaccines (Basel). 2021 Sep 17;9(9):1034. doi: 10.3390/vaccines9091034 (PMC8472562; doi:10.3390/vaccines9091034)
Supplement: Supplementary file 1 [file vaccines-09-01034-s001.zip › vaccines-1342239-supplementary.pdf]

vaccines

MDPI

Supplementary Materials

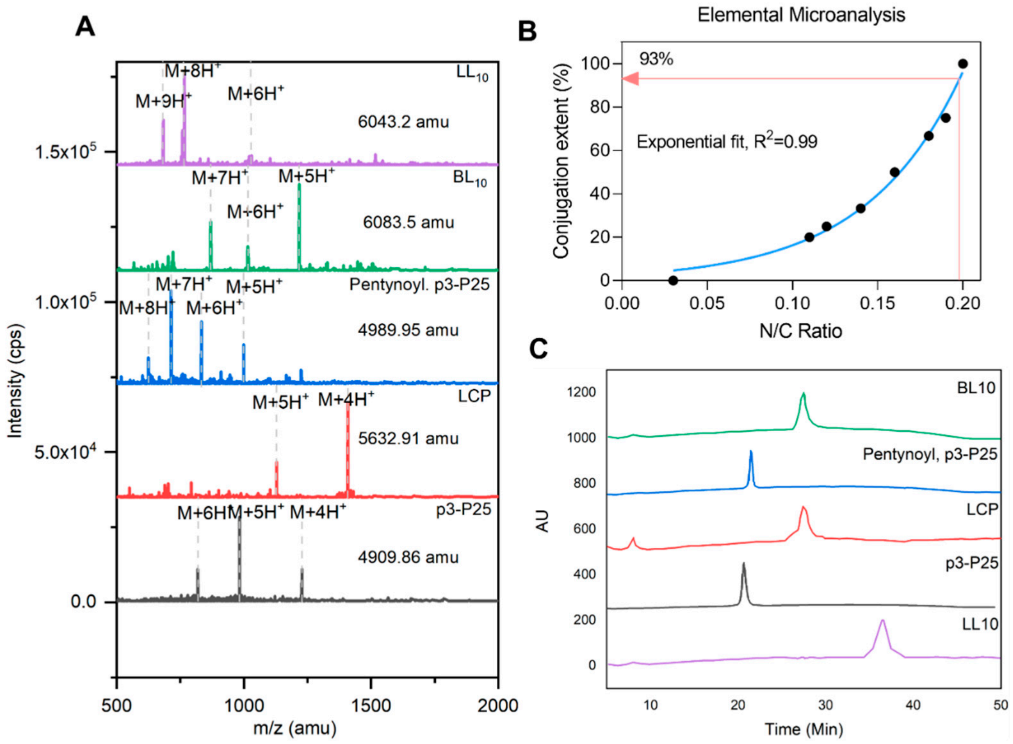

Figure S1. Physicochemical characterization of vaccine candidates: MS-ESI spectra (A); elemental analysis (B); and HPLC chromatograms (C). Typical signal broadening can be observed for amphiphile conjugates, LL10, BL10 and LCP.

Peak Analysis

Data Set:[Book1]Sheet1!I Date:5/15/2021
BaseLine:Constant
Chi^2=5.22539E-012 Adj. R-Square=9.98678E-001 # of Data Points=111
SS=3.39650E-010 Degrees of Freedom=65

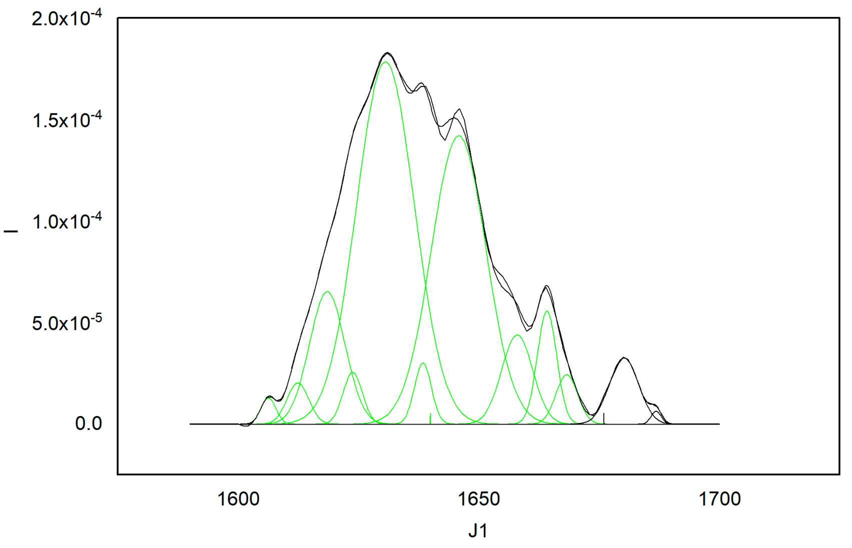

Fitting Results

Peak Index	Peak Type	Area Intg	FWHM	Max Height	Center Grvty	Area IntgP
1	Gaussian	4.9999E-5	3.66156	1.28281E-5	1606.31905	0.73581
2	Gaussian	1.16278E-4	5.40527	2.02091E-5	1612.42066	1.71121
3	Gaussian	6.06403E-4	8.71815	6.53439E-5	1618.52385	8.92417
4	Gaussian	1.28201E-4	4.71912	2.5521E-5	1623.81496	1.88668
5	Gaussian	0.00269	14.16908	1.78539E-4	1630.63017	39.62902
6	Gaussian	1.33073E-4	4.15645	3.00771E-5	1638.41399	1.95838
7	Gaussian	2.03523E-15	0.03159	5.44114E-6	1639.99134	2.99516E-11
8	Gaussian	0.00202	13.36437	1.4228E-4	1645.84426	29.78723
9	Gaussian	0	0	0	0	0
10	Gaussian	3.578E-4	7.61723	4.41277E-5	1658	5.26559
11	Gaussian	2.82157E-4	4.74909	5.58146E-5	1664.18163	4.15238
12	Gaussian	1.38522E-4	5.35344	2.43081E-5	1668.29967	2.03856
13	Gaussian	2.02023E-7	0.04292	5.43089E-6	1676.02879	0.00297
14	Gaussian	2.48312E-4	7.14342	3.26557E-5	1680.18416	3.6543
15	Gaussian	1.72386E-5	2.55992	6.32618E-6	1686.85776	0.25369

Figure S2. An example of secondary structure determination by FTIR-ATR.

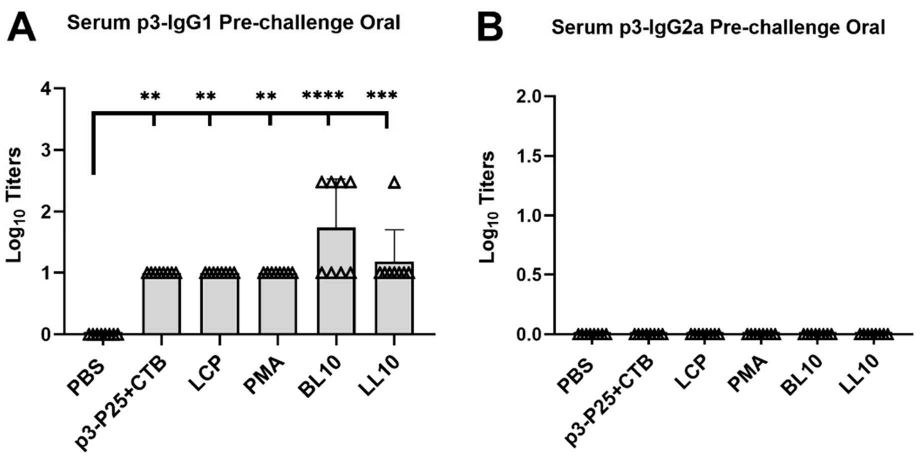

Figure S3. Subclasses of serum p3-IgG titers: A) serum p3-IgG1 titers, and B) serum p3-IgG2a titers. These demonstrate that the main IgG subclass is of the neutralizing IgG1 type. The horizontal dashed line represents the starting dilution.

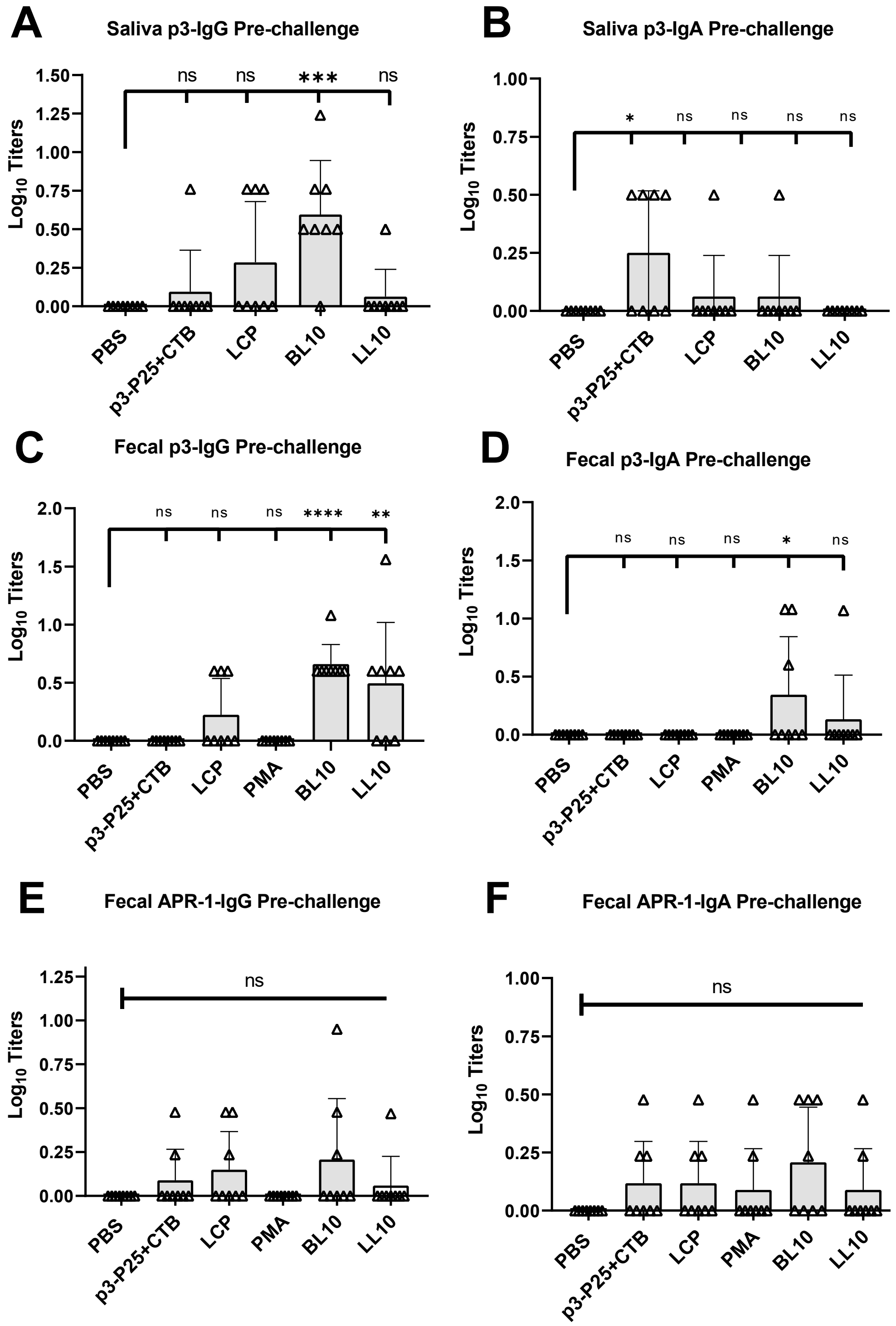

Figure S4. Pre-challenge salivary p3-IgG (A) and p3-IgA (B), fecal p3-IgG (C), fecal p3-IgA (D), fecal APR-1-IgG (E), and fecal APR-1-IgA (F) log10 titers.

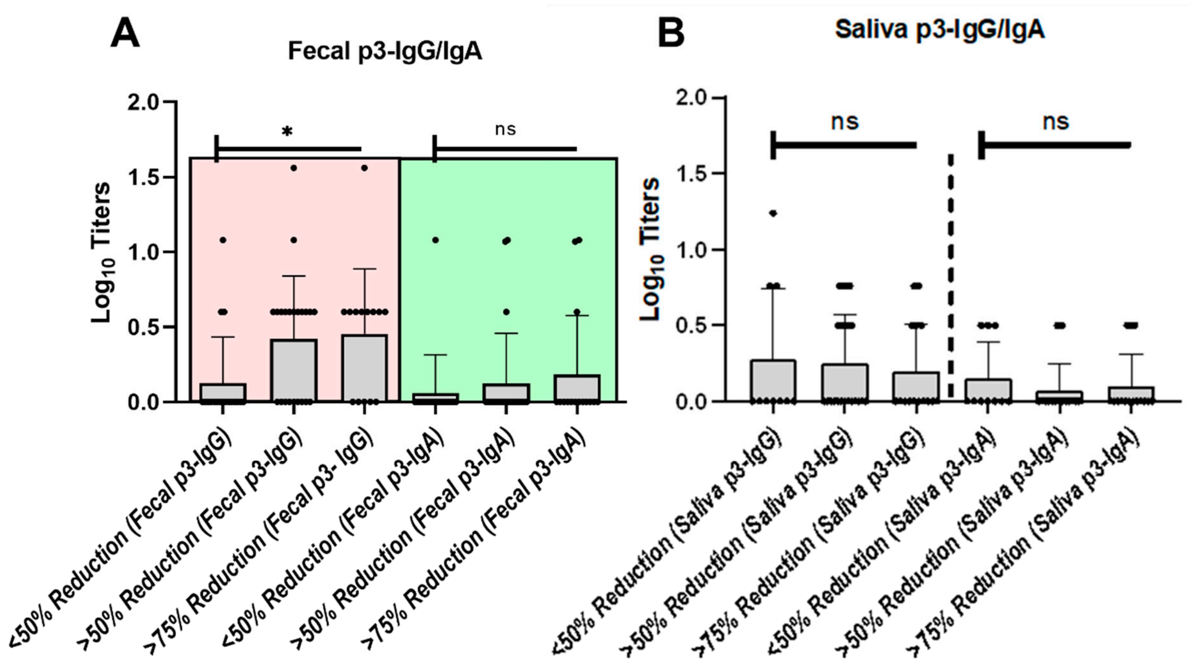

Figure S5. Protective capacity of fecal p3-IgG/IgA (A), and salivary p3-IgG/IgA titers (B).

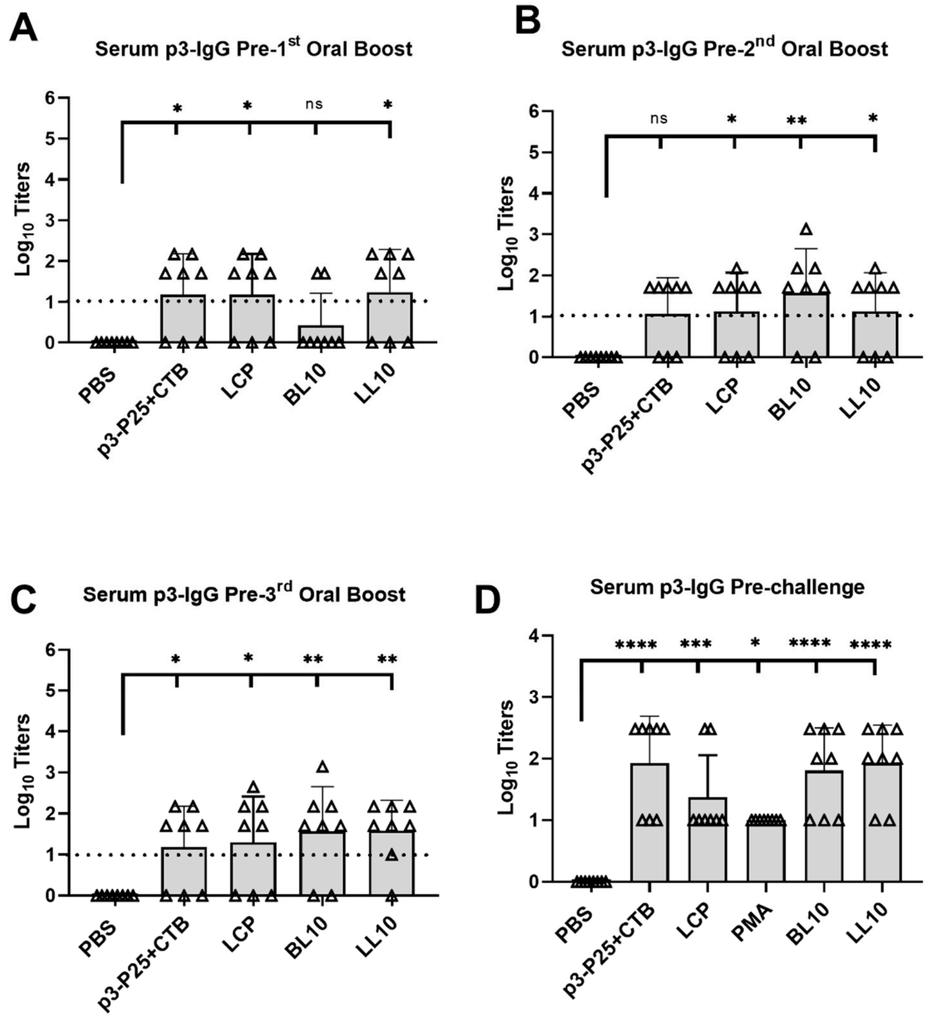

Figure S8. Serum anti-p3 IgG titers after 1st immunization (A), 2nd immunization (B), 3rd immunization (C), and 4th immunization (D).

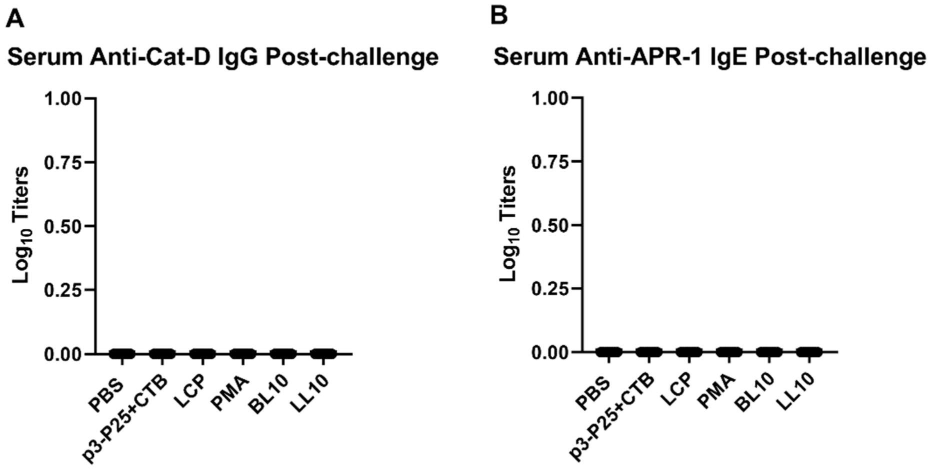

Figure S9. Serum anti-human cathepsin-D IgG titers (A), and serum anti-APR-1 IgE titers were both undetectable using post-challenge mice sera.

```
CLUSTAL 2.1 multiple sequence alignment


Mouse           MKTPGVLLLILGLLASSSFAIIRIPLRKFTSIRRTMTEVGGSVEDLILKGPITKYSMQ--
Human           MQPSSLLPLALCLLAAPASALVRIPLHKFTSIRRTMSEVGGSVEDLIAKGPVSKYSQA--
APR-1           -MARLVFLLVLCTLAAASVHRRLFHQARRHVTSVSLSRQPTLRERLIASGSWEDYQKQRY
                  .  :: * *  **:.:     :   :      :::.     * ** .*.  .*.

Mouse           ---------------SSPKTTEPVSELLKNYLDAQYYGDIGIGTPPQCFTVVFDTGSSNL
Human           ---------------VPAVTEGPIPEVLKNYMDAQYYGEIGIGTPPQCFTVVFDTGSSNL
APR-1           HYRKKILAKYAANKASKLQSANEIDELLRNYMDAQYYGVIQIGTPAQNFTVIFDTGSSNL
                                   :   : *:*:**:****** * ****.* ***:********

Mouse           WVPSIHCKILDIACWVHHKYNSDKSSTYVKNGTSFDIHYGSGSLSGYLSQDTVSVPCKSD
Human           WVPSIHCKLLDIACWIHHKYNSDKSSTYVKNGTSFDIHYGSGSLSGYLSQDTVSVPCQSA
APR-1           WVPSRKCPFYDIACMLHHRYDSGASSTYKEDGRKMAIQYGTGSMKGFISKDIVCIAG---
                **** :* : **** :**:*:*. **** ::* .: *:**:**:.*::*:* *.:.

Mouse           QSKARG--IKVEKQIFGEATKQPGIVFVAAKFDGILGMGYPHISVNNVLPVFDNLMQQKL
Human           SSASALGGVKVERQVFGEATKQPGITFIAAKFDGILGMAYPRISVNNVLPVFDNLMQQKL
APR-1           --------ICAEEQPFAEATSEPGLTFIAAKFDGILGMAFPEIAVLGVTPVFHTFIEQKK
                        : .*.* *.***.:**:.*:**********.:*.*:* .* ***..:::**

Mouse           VDKNIFSFYLNRDPEGQPGGELMLGGTDSKYYHGELSYLNVTRKAYWQVHMDQLEVGNEL
Human           VDQNIFSFYLSRDPDAQPGGELMLGGTDSKYYKGSLSYLNVTRKAYWQVHLDQVEVASGL
APR-1           VPSPVFAFWLNRNPESEIGGEITFGGVDTRRYVEPITWTPVTRRGYWQFKMDMVQGGSSS
                * . :*:*:*.*:*:.: ***: :**.*:: *   :::  ***:.***.::* :: ..

Mouse           TLCKGGCEAIVDTGTSLLVGPVEEVKELQKAIGAVPLIQGEYMIPCEKVSSLPTVYLKLG
Human           TLCKEGCEAIVDTGTSLMVGPVDEVRELQKAIGAVPLIQGEYMIPCEKVSTLPAITLKLG
APR-1           IACPNGCQAIADTGTSLIAGPKAQVEAIQKYIGAEPLMKGEYMIPCDKVPSLPDVSFIID
                  *  **:**.******:.**  :*. :** *** **::*******:**.:** : : :.

Mouse           GKNYELHPDKYILKVSQGGKTICLSGFMGMDIPPPSGPLWILGDVFIGSYYTVFDRDNNR
Human           GKGYKLSPEDYTLKVSQAGKTLCLSGFMGMDIPPPSGPLWILGDVFIGRYYTVFDRDNNR
APR-1           GKTFTLKGEDYVLTVKAAGKSICLSGFMGMDFPEKIGELWILGDVFIGKYYTVFDVGQAR
                ** : *  :.* *.*. .**::*********:*   * ********** ****** .: *

Mouse           VGFANAVVL-----------------------------
Human           VGFAEAARL-----------------------------
APR-1           VGFAQAKSEDGFPVGTPVRTFRQLQEDSDSDEDDVFTF
                ****:*
```

Figure S10. **Protein sequences alignment of mouse cathepsin-D, Human cathepsin-, and Na-APR-1, orange highlighted sequence showing lack of similarity of p3 epitope (APR-1) to corresponding sequences in mouse or human cathepsin-D sequences. Mouse cathepsin-D (UniProt P18242) sequence is 89% identical to human cathepsin (UniProt P07339), and only 46% identical to hookworm Na-APR-1 enzyme sequence (UniProt Q9N9H3) using CLUSTALW.**
